# Microalgal Co-Cultivation Prospecting to Modulate Vitamin and Bioactive Compounds Production

**DOI:** 10.3390/antiox10091360

**Published:** 2021-08-26

**Authors:** Luigi Pistelli, Angelo Del Mondo, Arianna Smerilli, Federico Corato, Concetta Piscitelli, Paola Pellone, Dora Allegra Carbone, Clementina Sansone, Christophe Brunet

**Affiliations:** Stazione Zoologica Anton Dohrn, Villa Comunale, 80121 Napoli, Italy; Luigi.pistelli@szn.it (L.P.); angelo.delmondo@szn.it (A.D.M.); arianna.smerilli@szn.it (A.S.); federico.corato@szn.it (F.C.); tina.piscitelli@virgilio.it (C.P.); paola.pellone@gmail.com (P.P.); doraallegracarbone@gmail.com (D.A.C.); christophe.brunet@szn.it (C.B.)

**Keywords:** microalgae, vitamins, bioactivity, polyphenols, antioxidant activity, biomass, blue biotechnology

## Abstract

Microalgal biotechnology is gaining importance. However, key issues in the pipeline from species selection towards large biomass production still require improvements to maximize the yield and lower the microalgal production costs. This study explores a co-cultivation strategy to improve the bioactive compounds richness of the harvested microalgal biomass. Based on their biotechnological potential, two diatoms (*Skeletonema marinoi*, *Cyclotella cryptica*) and one eustigmatophyte (*Nannochloropsis oceanica*) were grown alone or in combination. Concentrations of ten vitamins (A, B_1_, B_2_, B_6_, B_12_, C, D_2_, D_3_, E and H), carotenoids and polyphenols, together with total flavonoids, sterols, lipids, proteins and carbohydrates, were compared. Moreover, antioxidant capacity and chemopreventive potential in terms inhibiting four human tumor-derived and normal cell lines proliferation were evaluated. Co-cultivation can engender biomass with emergent properties regarding bioactivity or bioactive chemical profile, depending on the combined species. The high vitamin content of *C. cryptica* or *N. oceanica* further enhanced (until 10% more) when co-cultivated, explaining the two-fold increase of the antioxidant capacity of the combined *C. cryptica* and *N. oceanica* biomass. Differently, the chemopreventive activity was valuably enhanced when coupling the two diatoms *C. cryptica* and *S. marinoi*. The results obtained in this pilot study promote microalgal co-cultivation as a valuable strategy aiming to boost their application in eco-sustainable biotechnology.

## 1. Introduction

Microalgae are undoubtedly part of the future of eco-sustainable biotechnology, as a resource or source of biomass for the production of nutraceuticals or cosmeceuticals, as well as for processes related to wastewater treatments or bioenergy [1]. The same biological and ecological properties allowing them to be widespread microbes in all the aquatic environments [2,3], can be exploited in the biotechnological sector. Their small size determines a lower resource requirement compared to higher plants, while their unicellular nature makes them appealing for efficient large-scale production processes, being unnecessary the separation from waste and recalcitrant matter. Although the microalgal market is increasing all over the world [4,5], mainly thanks to only a few species (e.g., *Spirulina*, *Chlorella*, *Dunaliella*, or *Haematococcus*: [6]) and/or to some products (such as carotenoids, e.g., astaxanthin [7]), microalgal biotechnology is still in its infancy [8]. In addition, the promises—and premises—of microalgal biotechnology extending in different fields, the economic feasibility constitutes the main drawback of large-scale production [9,10,11]. Many studies highlight the need for further (bio-) technological improvements [12,13] aiming to lower the costs and/or to increase microalgal productivity. Improvements would regard different endpoints of the pipeline development for microalgal production. Firstly, the number of species used as a resource needs to be increased and diversified, targeting species with biological interests in terms of growth and richness of compounds produced. For this purpose, a gap of knowledge on some bioactive components in microalgae, such as vitamins or polyphenols content, is noteworthy [14,15]. Indeed, the bioactivity of these compounds is highly promising in terms of human health benefits [14,15,16]. An extended bioprospecting of microalgal biodiversity and chemodiversity, focusing on different families of compounds, is urgently required. A second aspect concerns the cultivation, aiming to lower the costs and/or enhance the quality or quantity of harvested biomass [12,13,17]. Indoor cultivation leads to a more controlled growth, conversely to outdoor microalgal cultivation strategy [18,19]. It allows the control of key variables, such as light, nutrient supply, CO_2_, temperature and mixing driving microalgal biomass yield. Recent findings highlighted the relevance in well-tuning light or mixing to enhance the microalgal productivity [10,19,20,21,22,23,24]. Asides from physical or chemical improvements, biological manipulation might be also undertaken. An example is represented by the co-cultivation of microorganisms [25]. Bacteria-microalga or yeast-microalga co-cultivation already showed promising results for biotechnological purposes [26,27], such as for the production of vitamins [14] and might represent a valuable road to both lower the costs and improve the production. The present study explores this overall strategy, with the aim to evince the advantages of co-cultivating two different microalgal species in producing an enriched bioactive biomass. If the co-cultivated species tolerate each other—as it occurs in nature while growing/competing for the same few resources, such as light or nutrients (the so-called “*plankton paradox*” [28])—it is expected that the harvested biomass shares the chemodiversity of the two species. However, it is not excluded that species interactions could lead to the synthesis of specific compounds. For instance, in terrestrial plants, polyphenols are involved in the biochemical response elicited by interspecific interactions [15,29]. Three marine microalgal strains with biotechnological potential were selected in this study, namely two Bacillariophyceae *Skeletonema marinoi* (CCMP 2092, S.m.) and *Cyclotella cryptica* (CCMP332, C.c.) and the Eustigmatophyceae *Nannochloropsis oceanica* (CCMP1779, N.o.). These species have biotechnological potential: S.m. is used as feed stock in aquaculture [30], while C.c. and N.o. are rich in lipids and promising as biofuel production sources [31,32]. N.o. is also investigated for human nutrition complements [33].

A comparative analysis of the macromolecular composition (proteins, lipids, carbohydrates), alongside with the vitamins content (A, B_1_, B_2_, B_6_, B_12_, C, D_2_, D_3_, E and H), total sterols, carotenoids, polyphenols and flavonoids concentrations among the mono- and the co-cultures was conducted. Antioxidant capacity was estimated with both ABTS and DPPH assays [34]. In vitro viability assays on three human tumor-derived and one normal cell lines were performed to assess the potential chemopreventive effect of the extracts from microalgal mono- or co-cultivation. This study investigates for the first time the almost complete vitamins profile together with the concentration of different families of bioactive compounds in microalgal biomass, challenging the role of co-cultivation in maximizing their yield.

## 2. Materials and Methods

### 2.1. Microalgal Species and Cultivation

Our study targeted three marine microalgal strains (obtained from the National Center for Marine Algae and Microbiota, Bigelow Laboratory, East Boothbay, ME, USA; Table S1): *Skeletonema marinoi* (CCMP 2092, S.m., Bacillariophyceae,), *Cyclotella cryptica* (CCMP332, C.c., Bacillariophyceae) and *Nannochloropsis oceanica* (CCMP1779, N.o., Eustigmatophyceae).

Microalgae were grown at 2 °C in flasks (50 to 250 mL) in autoclaved seawater, pre-filtered through a 0.7 µm GF/F glass-fiber filter and enriched with F/2 culture medium nutrients [22]. Cells were grown with a photoperiod of 12:12 dark:light under a sinusoidal white light distribution with a midday light intensity peak of 150 µmol photons s^−1^ m^−2^, following previous protocols and results [23,35,36]. White light, composed by Red:Green:Blue (10:40:50), was provided by a custom-built LED illumination system [37]. Light intensity was measured inside each flask with a PAR 4π sensor (QSL 2101, Biospherical Instruments Inc., San Diego, CA, USA), while the spectral composition was measured by using a spectroradiometer (Hyper OCR I, Satlantic, Halifax, CA, USA).

### 2.2. Experimental Strategy and Sampling

As a first step, six cultures—three monospecific and three couples of species—were grown in 50 mL flasks to test the co-cultivation potential and to assess the growth rate of each species. Daily microalgal counts were carried out to characterize the growth curve for each species. Once verified their compatibility, a scale-up was performed in 2 L carboys with air bubbling. All experiments were run in triplicate. The day after that the species—or at least one species in co-culture—reached the exponential growth phase, cells were exposed to a gradual 6 h-light increase from dark (dawn) to 600 µmol photons s^−1^ m^−2^ (midday) to boost the bioactive compound synthesis [24] and then collected at midday. Cells were centrifuged at 2000× *g* for 15 min at 4 °C (DR15P centrifuge, B. Braun Biotech International, Melsungen, Germany). Pellets were flash-frozen in liquid nitrogen and stored at −20 °C and subsequently lyophilized in a Freeze Dryer Modulyo (Edwards). Dry weight was accurately measured (dry weight, mg^-1^ DW). Antioxidant activity, bioactivity, macromolecular composition and bioactive compounds concentration of the biomass were then analyzed (Figure S1).

### 2.3. Cell Concentration and Microalgal Growth Rate

Cells were counted with a Zeiss Axioskop 2 Plus light microscope.; 1 mL of culture was added in a Sedgewick Rafter counting cell chamber. *Nannochloropsis oceanica* was counted using a Bürker counting chamber. Growth rate was estimated from cell concentration measurements using the following equation:

µ (d^−1^) = Ln(C_(*n*)_/C_(*n*-1)_)/(T_n_ − T_(*n*−1)_), (1)

where µ is the growth rate, C_(*n*−1)_ and C_n_ are cell concentrations (mL^−1^) at day T_(*n*−1)_ and day T*_n_*, respectively.

### 2.4. DPPH (2,2-Diphenyl-1-Picrylhydrazyl) Assay

An aliquot of 50 mg of dried algal powder was used to test the ability of the extract to scavenge 2,2-diphenyl-1-picrylhydrazyl (DPPH, CAS No. 1898-66-4, cat. No. 257621, Sigma-Aldrich, Saint Louis, MO, USA). Microalgal dried biomass was re-suspended in 500 μL of methanol and shacked vigorously for 2 min. The solution was sonicated for 1 min with a micro tip at 20% output on ice (S-250A Branson Ultrasonic, Brookfield, CT, USA) and then the tubes were centrifuged at 3900× *g* for 15 min at 4 °C. The supernatants were transferred into fresh tubes at a concentration of 100 μg mL^−1^ and then in a 96 well plate (transparent flat bottom, TPP Techno Plastic Products AG, Trasadingen, Switzerland), adding DPPH (previously dissolved in methanol) at a final concentration of 0.1 mM and kept for 30 min in the dark. The scavenging assay was performed in triplicate and the results were expressed as percentage reduction of the initial DPPH^+^ radical absorption at 517 nm using a Microplate Reader: Infinite^®^ M1000 PRO (TECAN, Männedorf, Switzerland). Results were calculated according to the equation: DPPH % Inhibition = [(A_0_ − A_1_)/A_0_] × 100, where A_0_ = absorbance of DPPH^+^ and A_1_= absorbance of sample.

### 2.5. ABTS (2,2′-Azinobis-3-Ethylbenzothiazoline-6-Sulphonic Acid) Assay

An aliquot of 50 mg of dried algal powder was used to test the ability of the extract to scavenge ABTS^+^ radical. Microalgal dry biomass was re-suspended in 500 μL of methanol and shacked vigorously for 2 min. The solution was sonicated for 1 min with a micro tip at 20% output on ice (S-250A Branson Ultrasonic) and centrifuged at 3900× *g* for 15 min at 4 °C. then, an aliquot of 10 μL of sample was mixed with 990 μL of an ABTS^+^ solution and kept for 6 min at 30 °C. The ABTS^+^ solution was prepared as follow: ABTS (CAS No. 30931-67-0, Sigma-Aldrich, St. Louis, MO, USA) was weighed and dissolved in water, with a final concentration of 7 mM. Potassium persulfate (CAS No. 7727-21-1, Sigma-Aldrich, St. Louis, MO, USA) powder was weighed and dissolved in water with a final concentration of 2.45 mM. To form the ABTS^+^ radical, an equal volume of ABTS and potassium persulfate solution was mixed and stored in darkness at room temperature for 12 h. The ABTS^+^ solution was then diluted with methanol to reach an absorbance of 0.70 ± 0.02 AU, measured in a UV-VIS spectrophotometer (UV/VIS Spectrophotometer Lambda 35, Perkin Elmer, Waltham, MA, USA) at 734 nm. After incubation of the sample with ABTS^+^ solution, the absorbance was read at 734 nm using a UV-VIS spectrophotometer (UV/VIS Spectrophotometer Lambda 35, Perkin Elmer). The scavenging assay was done in triplicate and the results were calculated according to the equation: ABTS^+^ % Inhibition = [(A_0_ − A_1_)/A_0_] × 100, where A_0_= absorbance of ABTS^+^ and A_1_= absorbance of sample.

### 2.6. Total Carbohydrate Content

Total carbohydrate concentration was determined on 30 mg of dried algal powder with the phenol-sulfuric acid method, slightly modified from [38]. The dried biomass was re-suspended in 1.8 mL of 1 M sulfuric acid and heated at 95 °C for 2 h. After cooling at room temperature, the tubes were centrifuged (14,000× *g* for 10 min). Phenol (0.2 mL) was added to 0.4 mL of sample, then 1 mL of concentrated sulfuric acid was added. After 30 min at room temperature, the absorbance was read at 490 nm using a UV-VIS spectrophotometer (UV/VIS Spectrophotometer Lambda 35, Perkin Elmer). The total carbohydrates concentration was quantified referring to a calibration curve using glucose as standard.

### 2.7. Total Protein Content

Dried microalgal powder (15 mg) was re-suspended in 500 µL of RIPA Lysis and Extraction Buffer (Thermo Fisher Scientific, Waltham, MA, USA) and sonicated for 90 s (three pulses of 30 s with 30 s intervals between each pulse) with a micro tip at 20% output on ice (S-250A Branson Ultrasonic). Samples were then centrifuged at 13,000× *g* for 8 min. The absorbance of an aliquot of 1.5 µL of the supernatant was read in triplicate in a NanoDrop 1000 UV-Vis spectrophotometer at 280 nm and 260 nm to estimate the protein concentration [39].

### 2.8. Total Lipid and Sterol Content

For total lipid concentration estimation [40], 100 mg of dried microalgal powder was dissolved in 4 mL of methanol. Chloroform (2 mL) and water (0.4 mL) were then added and the mixture was vortex-mixed for 30 s. Then, chloroform (2 mL) and water (2 mL) were added and the mixture was vortex-mixed for additional 30 s. The tubes were centrifuged at 600× *g* for 10 min. After discarding the upper layer, the lower layer was transferred into a fresh tube and the pellets were re-extracted in a solution of chloroform (2 mL) and methanol (2 mL). The extract was passed through a layer of anhydrous sodium sulfate using Whatman No. 1 filter paper in a funnel and then dried by rotary evaporation at 4 °C and weighted. The dried total lipid content was used for total sterol concentration (TSC) estimation [41]. The lipid extract was re-suspended in 2.5 mL of chloroform, then 800 µL of sample were mixed with 400 µL of Liebermann-Burchard reagent and incubated at 3 °C for 30 min. The Liebermann–Burchard reagent, prepared in ice water, was composed by 30% glacial acetic acid, 60% acetic anhydride and 10% sulfuric acid. Anhydrous sodium sulfate was added to the mixture at a final a concentration of 2%. The absorbance was read at 617 nm, using a UV-VIS spectrophotometer (UV/VIS Spectrophotometer Lambda 35, Perkin Elmer) and the sterol concentration was quantified thanks to a calibration curve using cholesterol as standard.

### 2.9. Total Polyphenol Content and Total Flavonoid Content

The total phenolic content (TPC) and total flavonoid content (TFC) of the microalgal biomass were estimated in aliquots of 30 mg of dried powder. An aliquot of 150 μL of the extract was mixed with 750 µL of Folin-Ciocalteu’s phenol reagent, pre-diluted in distilled water 1:10 *v*/*v*. After 4 min, 600 μL of Na_2_CO_3_ (75 g L^−1^) were added to the mixture and left 2 h at room temperature. Then, absorbance was read at 765 nm using a UV-VIS spectrophotometer (UV/VIS Spectrophotometer Lambda 35, Perkin Elmer) and the concentrations were estimated using gallic acid (GA) as standard [24]. TFC was estimated by the aluminum chloride (AlCl_3_) colorimetric method [24] as follows: an equal volume of AlCl_3_ 2% was mixed to 600 μL of sample pre-diluted 1:2 *v*/*v* in methanol 80% *v*/*v*. The mixture was left for 1 h at room temperature. Then, absorbance was read at 410 nm using a UV-VIS spectrophotometer (UV/VIS Spectrophotometer Lambda 35, Perkin Elmer) and the concentrations were estimated using quercetin (Q) as standard.

### 2.10. HPLC Analysis of Pigments

Pigment analysis was done following the protocol in [24]. Fifteen mg of dried algal powder were placed in 2 mL of absolute methanol and pigments were extracted by mechanical grounding for 3 min. After filtration of the homogenate onto Whatman 25 mm GF/F filters, the volume of the extract was accurately measured. An aliquot of 500 µL of the pigment extract was mixed to 250 µL of 1 M ammonium acetate and incubated for 5 min in darkness at 4 °C. Injection of the extract in a Hewlett Packard series 1100 HPLC, equipped with a reversed-phase column (2.6 mm diameter C8 Kinetex column; 50 × 4.6 mm.; Phenomenex, Torrance, CA, USA) was carried out through a 50 µL loop. The mobile phase was composed of two solvents mixtures: A, methanol:0.5 N aqueous ammonium acetate (70:30, *v*/*v*) and B, absolute methanol with a flow rate set up at 1.7 mL min^−1^. During the 12 min elution, the gradient between the solvents was programmed as follow: 75% A (0 min), 50% A (1 min), 0% A (8 min), 0% A (11 min), 75% A (12 min). Pigments were detected at 440 nm using a Hewlett Packard photodiode array detector model DAD series 1100, which provides the 400–700 nm spectrum for each detected pigment. Pigments were identified based on their retention time and absorption spectrum, while pure standards from the D.H.I. Water & Environment (Hørsholm, Denmark) allowed their quantification.

### 2.11. Vitamin Determination

Dried microalgal powder (15 mg) was re-suspended in 500 µL of RIPA Lysis and Extraction Buffer (Thermo Fisher Scientific, Waltham, MA, USA) and sonicated for 90 s (three pulses of 30 s with 30 s intervals between each pulse) with a micro tip at 20% output on ice (S-250A Branson Ultrasonic). The samples were then centrifuged at 13,000× *g* for 8 min. Vitamins A, B_1_, B_2_, B_6_, B_12_, C, D_2_, D_3_, E and H were quantified performing a competitive ELISA assay in 96 well plate (transparent flat bottom, TPP Techno Plastic Products AG, Trasadingen, Switzerland): 5 µL of each microalgal extract, 45 µL of coating buffer solution (0.05 M Carbonate-Bicarbonate, pH = 9.6) and 50 µL of primary antibody solution (primary antibody 1:1000 dilution in TBS + 5% BSA + 0.05% Tween20^®^.; Table S2) were added in each well and incubated at 37 °C for 1 h. After the incubation, 50 µL of secondary antibody solution (Goat α-Rabbit HRP conjugated antibody 1:250 dilution in TBS + 5% BSA + 0.05% Tween20^®^) were added in each well and incubated at 37 °C for 1 h. At the end of the second incubation, 50 µL of TMB substrate 1× (Abcam, Cambridge, UK) were added in each well and incubated at room temperature for 15 min. The absorbance was read at 450 nm with the reference measurement at 620 nm, using a Microplate Reader: Infinite^®^ M1000 PRO (TECAN, Männedorf, Switzerland). The vitamin concentration (in ng mg^−1^ DW) was quantified referring to calibration curve using pure vitamins as standard.

### 2.12. HPLC Analysis of Phenolic Compounds

An aliquot of around 130 mg was used to determine the phenolic compounds profile of the dried microalgal powder. Extraction was done in 2 mL of 75% aqueous methanol, with BHT (butylated hydroxytoluene, 1% final) to preserve polyphenol stability. Extraction was performed with ultrasounds for 2 min followed by a 30 min lasting ultrasonic bath. Then, the mixture was kept for 30 min at 4 °C. Extracts were then centrifuged at 1200× *g* for 5 min and the supernatants were filtered on a 0.2 µm polyamide membrane. The supernatant was then injected in a Hewlett Packard series 1100 HPLC (Hewlett-Packard, Wilmington, NC, USA) equipped with a 50 µL loop and a reversed-phase column (5 µm diameter C18 Kinetex column.; 250 × 4.6 mm.; Phenomenex, USA). The mobile phase, as described in [42], consisted of 1% aqueous acetic acid solution (A) and 100% methanol (B) with the following gradient: 90% A from 0 to 27 min, from 90 to 60% A in 28 min, 60% A for 5 min, from 60 to 56% A in 2 min, 56% A for 8 min, from 56 to 90% A in 1 min and 4 min 90% A. The flow rate was constant at 1 mL min^−1^. Chromatograms were acquired at three different wavelengths (264, 278 and 310 nm). Phenolic compounds were identified based on their retention time and absorption spectrum (registered between 220 and 450 nm) and with comparison with nineteen pure standards (Merck KGaA, Darmstadt, Germany) allowing the quantification of these compounds. Due to experimental problems, the phenolic compounds analysis provided reliable results only for the mono-cultivated S.m. and C.c.

### 2.13. Human Cell Lines and MTT (3-(4,5-Dimethylthiazol-2-yl)-2,5-Diphenyltetrazolium Bromide) Assay

Four human cell lines were used to perform the MTT assay: prostate cancer (PC3; ATCC number: CRL-1435), melanoma (A2058; ATCC number: CRL-11147), colon-rectal adenocarcinoma (HT-29; ATCC number: HTB-38) and normal prostate epithelium (PNT2; ECACC 95012613). The cell lines A2058 and HT29 represent primary tumor cells, while PC3 cell line derived from prostate cancer metastasis in bone marrow in a disease state IV. PNT2 cell line was established by immortalization of normal adult prostatic epithelial cells by transfection with a plasmid containing SV40 genome with a defective replication origin. The PNT2 cell line was grown in RPMI 1640 supplemented with 10% (*v*/*v*) fetal bovine serum (FBS), 100 units mL^−1^ penicillin, 100 units mL^−1^ streptomycin and 2 mM of L-glutamine, in a 5% CO_2_ atmosphere at 37 °C. The PC3 cell line was grown in RPMI 1640 supplemented with 10% (*v*/*v*) FBS, 100 units mL^−1^ penicillin, 100 units mL^−1^ streptomycin and 2 mM of L-glutamine, in a 5% CO_2_ atmosphere at 37 °C. The A2058 cell line was grown in DMEM/F12 (Dulbecco’s Modified Eagle Medium/Nutrient Mixture F-12), supplemented with 10% FBS, 100 units mL^−1^ penicillin and 100 units mL^−1^ streptomycin, in a 5% CO_2_ atmosphere at 37 °C. The HT-29 cell line was grown in McCoy’s 5a Medium Modified supplemented with 10% (*v*/*v*) FBS, 100 units mL^−1^ penicillin and 100 units mL^−1^ streptomycin, in a 5% CO_2_ atmosphere at 37 °C. Human cell lines were seeded in 96 well plate (transparent flat bottom, TPP Techno Plastic Products AG, Trasadingen, Switzerland), with an initial concentration of 2 × 10^3^ cells well^−1^ and kept overnight for attachment. The four human cell lines were then incubated with microalgal extracts at three concentrations (1, 10 or 100 μg mL^−1^) for 48 h. Microalgal extracts were prepared as follow: Microalgal dried biomass was re-suspended in 500 μL of methanol and shacked vigorously for 2 min. The solution was sonicated for 1 min with a micro tip at 20% output on ice (S-250A Branson Ultrasonic) and then the tubes were centrifuged at 3900× *g* for 15 min at 4 °C. The supernatants were then dried by rotary evaporation at 37 °C and the dried methanolic extracts were resuspended in DMSO (0.1%) and human cell culture medium. At the end of the 48 h incubation, the MTT (3-(4,5-dimethylthiazol-2-yl)-2,5-diphenyltetrazolium Bromide, Applichem, Darmstadt, Germany) viability assay was performed. Cells were incubated with 10 μL (5 mg mL^−1^) of MTT for 3 h in a 5% CO_2_ atmosphere at 37 °C. The formazan crystals (produced by viable cells) were dissolved with 100 μL of isopropyl alcohol. The absorbance was then recorded on a microplate reader at a wavelength of 570 nm Infinite^®^ M1000 PRO (TECAN, Männedorf, Switzerland). The results were represented as a percent of viable cells estimated as the ratio between the absorbance of each sample (human cell line treated with microalgal extracts) and the absorbance of the control (untreated cells).

### 2.14. Statistical Analysis

For each data measured on the triplicate cultivations, mean and standard deviation were estimated using GraphPad^®^ Software (2021). Mean comparison was carried out using the Student’s *t*-test. For viability assay, two-way ANOVA was used for the assessment of variance within and between the control and treated groups. Dunnett’s method was applied to compare the mean from treated cells with the control mean (untreated cells) by using GraphPad^®^ Software (2021). A confidence interval at 95% for the difference between the two means was estimated.

## 3. Results

### 3.1. Growth and Biomass

Coupling two species together did not inhibit growth of the co-cultivated species, both in 50 mL flasks (Figure S2) and in 2 L flasks (Table 1). Growth rate tended to decrease when two species were coupled and the co-cultivation favored the two diatoms, S.m. or C.c. compared to N.o. (Table 1). This resulted in a low contribution (<15%) of N.o. to the total harvested biomass (Table 1), even though the N.o. cell concentration was higher than the diatoms cell concentration when cultivated together, N.o. being smaller than the other two species (Table S1).

### 3.2. ABTS and DPPH Scavenging Ability

**Figure 1:**
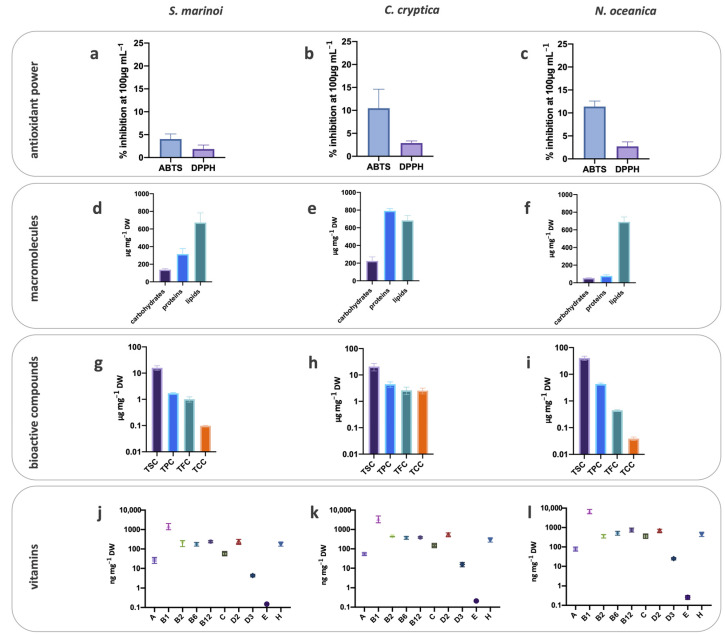
Antioxidant capacity (ABTS and DPPH scavenging ability) of the biomass from S.m. (**a**), C.c. (**b**) and N.o. (**c**); macromolecular composition of the biomass from S.m. (**d**), C.c. (**e**) and N.o. (**f**); bioactive compounds content of the biomass from S.m. (**g**), C.c. (**h**) and N.o. (**i**) and vitamin content of the biomass from S.m. (**j**), C.c. (**k**) and N.o. (**l**) (y axis in log scale). S.m. = *Skeletonema marinoi*, C.c. = *Cyclotella cryptica*, N.o.= *Nannochloropsis oceanica*. TSC = total sterols content, TCC = total carotenoids content, TPC = total polyphenols content, TFC = total flavonoids content. Values are expressed as mean ± SD.

**Figure 2:**
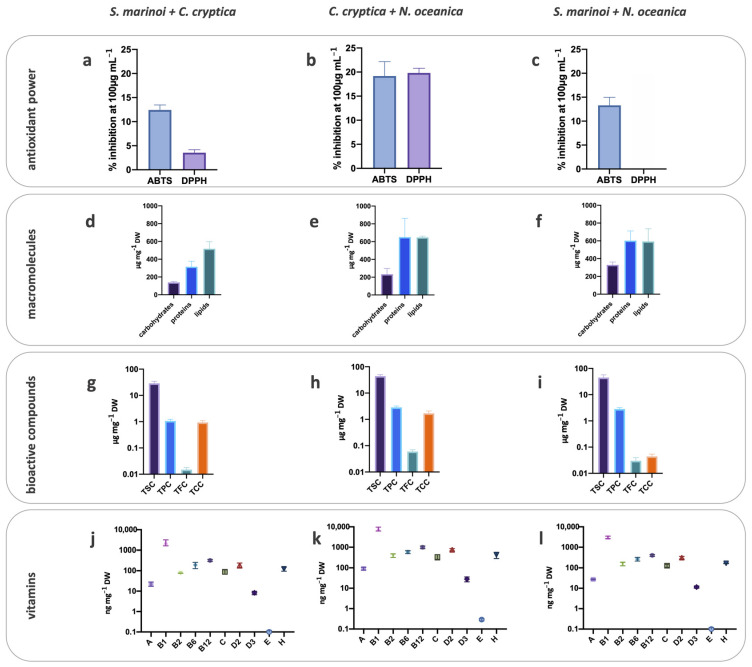
Antioxidant capacity (ABTS and DPPH scavenging ability) of the biomass from S.m. + C.c. (**a**), C.c. + N.o. (**b**) and S.m. + N.o. (**c**); macromolecular composition of the biomass from S.m. + C.c. (**d**), C.c. + N.o. (**e**) and S.m. + N.o. (**f**); bioactive compounds content of the biomass from S.m. + C.c. (**g**), C.c. + N.o. (**h**) and S.m. + N.o. (**i**) and vitamin content of the biomass from S.m. + C.c. (**j**), C.c. + N.o. (**k**) and S.m. + N.o. (**l**) (y axis in log scale). S.m. = *Skeletonema marinoi*, C.c. = *Cyclotella cryptica*, N.o. = *Nannochloropsis oceanica*. TSC = total sterols content, TCC= total carotenoids content, TPC = total polyphenols content, TFC= total flavonoids content. Values are expressed as mean ± SD.

The ABTS scavenging activity of S.m. was significantly lower than C.c. and N.o. (5% vs. 11 and 13%, respectively; *p* < 0.05; Figure 1a–c), while the DPPH scavenging ability was similar among the three species (Figure 1a–c). Generally, co-cultivation tended to increase the ABTS radical scavenging of the harvested biomass, compared to mono-cultures (Figure 2a–c). The two species, C.c. and N.o., cultivated together significantly strengthened the ABTS scavenging ability (Figure 2, *p* < 0.05). This result was confirmed by the significant enhancement of the DPPH scavenging ability for the biomass C.c. + N.o. compared to the other biomasses (*p* < 0.001; Figure 2a–c).

### 3.3. Macromolecular Composition

The macromolecular composition differed among the three species (Figure 1d–f). Protein content was the highest in C.c. compared to S.m. (two folds lower, *p* < 0.001) and N.o. (ten folds lower, *p* < 0.001). The same trend was found for the carbohydrate content, two and four folds higher in C.c. than in S.m. (*p* < 0.01) and N.o. (*p* < 0.001), respectively (Figure 1d–f). Lipid concentration was similar in all the three species (*p* > 0.05), with N.o. accounting for the greatest lipid contribution (84%, Figure 1d–f). Co-cultivation slightly modified this scenario, especially with N.o. (Figure 2d–f). The co-cultivation of a diatom (S.m. or C.c.) with N.o. enhanced the protein content in the harvested biomass compared to the co-cultivation without N.o. (*p* < 0.05). Conversely, the lipid content decreased when N.o. was coupled with S.m. or C.c. compared to N.o. alone (39 or 52%).

### 3.4. Bioactive Compounds

The richness in sterols (TSC= total sterols content), carotenoids (TCC = total carotenoids content), polyphenols (TPC= total polyphenols content) and flavonoids (TFC = total flavonoids content) differed among the biomass obtained from the three cultivated species (Figure 1g–i). N.o. displayed a higher TSC than C.c. or S.m. (*p* < 0.05) and the lowest TFC or TCC (*p* < 0.05) (Figure 1g–i).

C.c. exhibited a higher TCC than the two other species (*p* < 0.01). Indeed, the common pigments in the three species, chlorophyll *a* and β-carotene, were significantly higher in C.c. biomass than in the two other species (*p* < 0.05; data not shown). TFC was also higher in C.c. biomass (*p* < 0.01) (Figure 1g–i), while the highest TPC was recorded in C.c. and N.o. The phenolic compounds analysis—with reliable results only for the mono-cultivated S.m. and C.c.—revealed the dominance of gallic-like, sinapic acid, daidzein and genistein in both species, while p-coumaric acid and apigenin were only detected in C.c. and rutin only in S.m. (Table S3).

Coupling N.o. with diatoms provided the biomass with the highest TSC (*p* < 0.05, Figure 2g–i), with values almost similar to those obtained in the biomass from mono-cultivated N.o (Figure 1i). Whatever the co-cultivation, TPC and TFC lowered compared to the respective mono-cultures (*p* < 0.05, Figure 1g–i and Figure 2g–i), with a notable decrease of TFC (Figure 2g–i). Conversely, TCC in the harvested biomass from co-cultivation ranged between the content of the two species separately cultivated. Among carotenoids, zeaxanthin (the photoprotective pigment of N.o.) was retrieved in biomass from N.o. co-cultivation (N.o. + S.m. and N.o. + C.c., 0.010 ± 0.004 and 0.058 ± 0.02 µg mg^−1^ DW, respectively), while absent in mono-cultivated N.o. Conversely, no difference in diatoxanthin (Dt, the photoprotective pigment of S.m. and C.c.) nor in diadinoxanthin (Dd, the precursor of Dt) between co-cultivation and mono-cultivation was revealed (*p* > 0.05, data not shown). However, the de-epoxidation state (DES = Dt/(Dt + Dd); [43]) was significantly higher in S.m. than in C.c. (*p* < 0.05), both in mono- and co-cultures.

### 3.5. Vitamins

The ten analyzed vitamins were detected in the microalgal biomasses, with concentrations ranging from 0.10 to 8000 ng mg^−1^ DW (Figure 1j–l). Vitamins B_1_, B_2_, B_6_, B_12_, H (B_7_) and D_2_ were the most concentrated in all harvested biomasses from mono-cultivation (Figure 1j–l), while vitamins D_3_ and E were the lowest. N.o. biomass displayed the highest content of almost all vitamins (Figure 1j–l), with significant differences with S.m. and C.c. for the vitamins B_1_, B_12_, C and D_3_ (at least *p* < 0.05) and only with S.m. for B_2_, B_6_, H, D_2_, E and A (at least *p* < 0.05). The lowest vitamin concentration (Figure 1j–l and Figure 2j–l) was found in S.m. biomass, either mono- or co-cultivated.

The co-cultivation of C.c. + N.o. (the two species with the higher vitamin concentration reported from mono-culture) increased the concentration of the vitamins A, E, C, B_1_, B_12_ and B_6_ (at least *p* < 0.05) compared to C.c. alone. Generally, this co-cultivation enhanced the vitamin content (Figure 2j–l), the latter being significantly higher than in the two other co-cultures (S.m. + N.o. or S.m. + C.c.; at least *p* < 0.01).

### 3.6. Bioactivity of the Microalgal Extracts on Human Cells Viability

**Figure 3:**
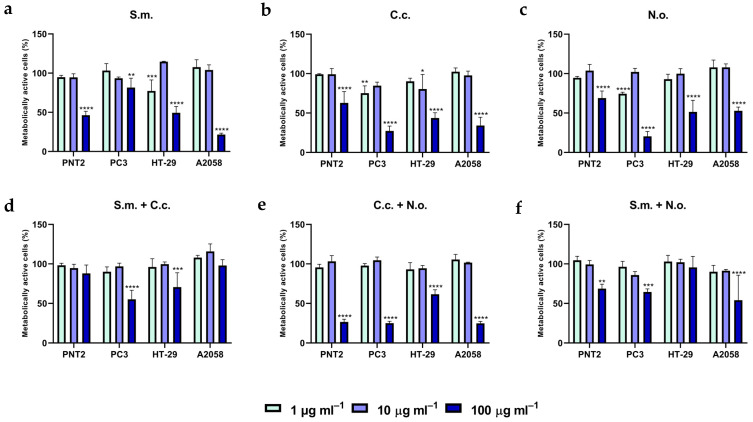
Percentage of viable human cells treated during 48 h with 1, 10 or 100 µg mL^−1^ of algal extract from mono and co-cultivation. Panels (**a**–**c**) represent results obtained by MTT assay on the PNT2, PC3, HT29 and A2058 cell lines treated with extracts from mono-cultivation. Panels (**d**–**f**) represent results obtained by MTT assay on the PNT2, PC3, HT29 and A2058 cell lines treated with extracts from co-cultivation. Statistical significance is calculated through Dunnett test and is indicated by **** (*p* ≤ 0.0001), *** (*p* ≤ 0.0005), ** (*p* ≤ 0.005) and * (*p* ≤ 0.05). S.m. = *Skeletonema marinoi*, C.c. = *Cyclotella cryptica*, N.o. = *Nannochloropsis oceanica*, S.m.+ C.c. = co-cultivation of C.c. and S.m., C.c. + N.o. = co-cultivation of C.c. and N.o., S.m. + N.o. = co-cultivation of S.m. and N.o. Values are expressed as mean ± SD.

The extracts from mono-cultures (Figure 3a–c and Figure S3) displayed a significant cytotoxicity (*p* < 0.0001) at the highest concentration (100 µg mL^−1^) on both cancer and normal cell lines. The two lower concentrations (1 and 10 µg mL^−1^) exhibited different effects on the human cell lines. At the lowest concentration (1 µg mL^−1^), S.m. extract induced an antiproliferative effect on the colon-rectal adenocarcinoma cell line (HT29, primary tumor cells), with a cell viability lowering at 77% (Figure 3a; *p* < 0.0005), while it did not affect normal prostate epithelium cell line (PNT2) viability. Differently, the lowest concentration (1 µg mL^−1^) of C.c. extract decreased the prostate cancer cell line (PC3, cancer disease state IV) viability to 75% (Figure 3b; *p* < 0.001) without compromising the PNT2 normal cell viability. As for C.c., N.o. extract (1 µg mL^−1^) displayed an antiproliferative effect on the PC3 cell line without affecting the PNT2 cell growth (Figure 3c; *p* < 0.0001).

Co-cultivation modified the antiproliferative capacity of the biomass extracts (Figure 3d–f and Figure S3). At the highest concentration, the S.m + C.c. extract enhanced the cytotoxicity on the PC3 cells (55% cell viability, *p* < 0.0001) (Figure 3d) compared for instance to S.m. in mono-culture (75% cell viability, Figure 3a). Moreover, S.m + C.c. extract did not affect the normal prostate epithelium (PNT2) cell line viability at none of the three concentrations tested (Figure 3d). Regarding the C.c. + N.o. co-cultivation, the highest concentration (100 µg mL^−1^) of this extract decreased the viability of all cell lines (Figure 3e; *p* < 0.0001), although no effect was reported with the two other concentrations (1 or 10 µg mL^−1^, Figure 3e; *p* > 0.05). S.m. + N.o. extract inhibited the human cell line A2058 viability at a concentration of 100 µg ml^−1^ (54%, Figure 3f; *p* < 0.0001), although the antiproliferative effect against the other cell lines was lower compared to S.m. or N.o. extracts from mono-cultivation.

## 4. Discussion

This study provides a broad integration of biochemical properties and bioactivities of biomass harvested from microalgae cultivated alone and in co-culture, also comparing their growth performance. The findings highlight the benefits of microalgal co-cultivation as a tool to produce enriched biomass with emergent biological properties, enhancing biotechnological interests, without increasing cultivation costs. This might represent a way to improve the microalgal role as a resource for human-health-applied biotechnology. The main advantage offered by the co-cultivation strategy is its capacity to enhance the bioactivity interest of the biomass, with an enrichment in vitamins and other bioactive molecules and/or in improving the bioactive properties of the biomass. The augmentation of the vitamins content is extremely appealing, being these compounds crucial for human health [14,44,45]. Although algae can be auxotroph for vitamins B_1_, H and B_12_ [19], their content in the harvested microalgal biomass is much higher than the quantity added in the F/2 medium (e.g., [22]), suggesting a capability of these microalgae to synthetize them [14]. This feature is relevant and further promotes microalgae as a valuable source of vitamins and amongst them, those generally low or absent in terrestrial plants (i.e., vitamins D and B).

The three species, i.e., the two Bacillariophyceae *Skeletonema marinoi* and *Cyclotella cryptica* and the Eustigmatophyceae *Nannochloropsis oceanica,* are able to grow in couple without generating competition-related growth inhibition. Competition for resources, light or nutrients, does not significantly affect the co-existence of the species, as it occurs in situ, with the co-occurrence of many species in a small habitat (the so-called “*plankton paradox*” [28]), probably allowed by different eco-physiological requirements. Regardless of the species, the three co-cultivations lower the biomass concentration in polyphenols and flavonoids, suggesting that the intracellular content of these compounds is modulated by the interactions between the species. It can be hypothesized the release in the medium of such compounds which are well-known allelochemical molecules [29,46,47]. The non-inhibition of the growth of the coupled species indicates that an autocrine signaling process might be induced, generating physiological adjustments of the species (e.g., [48]). Indeed, the detection of zeaxanthin—the photoprotective xanthophyll in N.o.—in the biomass derived from N.o. co-cultivation (N.o. + S.m. or N.o. + C.c.) while absent in biomass from N.o. mono-cultivation might confirm this feature, since zeaxanthin increase was already observed in response to allelochemicals in microalgae [49].

The results obtained in our study confirms the vitamin richness of *Nannochloropsis* [14]. Coupling N.o. with the diatom *Cyclotella cryptica* further increases the overall vitamin content together with the high phytosterols and carotenoids concentration. The increase in all vitamins reaches at least 10%, except for the vitamins C and H (5 and 2% lower), while carotenoids increase by 97% and sterols by 8%. The enrichment of the biochemical profile of N.o. + C.c. positively modulates the bioactive property of this mix, increasing its antioxidant capacity. Yet, the antiproliferative activity of N.o. + C.c. extract is enhanced when added at 100 µg mL^−1^ to the three human cancer cell lines, compared to the extracts from other cultures. Thishighlights the improved beneficial chemopreventive activities of the couple N.o. + C.c.. Vitamins or carotenoids—both enhanced in N.o. + C.c.—activities against cancer cells are well documented [50,51,52]. Instead, hypothetically, the reported cytostatic effect on the PNT2 cell line might be ascribed to the bioactive compounds-induced inhibition of SV40 (Simian virus) transfected in this cell line to render them high proliferative [53,54]. Indeed, the PNT2 cell proliferation is triggered by virus replication [55], the latter being favored by oxidative stress [56]. The richness in vitamins of the C.c. + N.o. biomass matches with its antiproliferative effect on PNT2 cells, since antioxidant molecules such as vitamins D, E, B_12_ or B_6_, among the others, are known to interfere with intracellular viral replication [57,58]. This hypothesis needs to be deeply investigated to be confirmed or refuted. In addition, the emerging bioactive property of the S.m. + C.c. biomass is strongly attractive. Indeed, it lowers the proliferation of the resistant and metastatic PC3 cell line, derived from bone metastatic prostate cancer, which exhibits drug resistance [59], without affecting PNT2 cell line viability. The comparison of the antioxidant activity and biochemical profile of C.c. + S.m. with the other (co-)cultivations does not fully explain its bioactive property. This result highlights the different type of activities between S.m. + C.c. and C.c. + N.o. biomass, also confirmed by the fact that vitamins, carotenoids and sterols contents are lower in S.m. + C.c. than in C.c. + N.o. co-culture. Hypothetically, the emerging bioactivity of C.c. + S.m. biomass might be relied to some specific compounds that enhance the bioactivity against PC3 cells proliferation, for instance polyphenols [60,61,62].

## 5. Conclusions

This pioneer study opens a new route to produce microalgal biomass with an increased bioactive compounds richness, e.g., vitamins, or/and an improved the bioactive property. Coupling two species with alternative biochemical or nutraceutical profile can improve the yield of vitamins and the antioxidant or chemopreventive properties of the biomass. The high vitamin content detected in *C. cryptica* and *N. oceanica* is enhanced by 10% when these two species are coupled, further increasing the antioxidant capacity of the biomass (almost doubled compared to the biomass from microalgal mono-culture). The antioxidant property enhancement leads to a significant increase in the antiproliferative capacity against the three cancer cell lines tested. The synergy of the two diatoms *C. cryptica* and *S. marinoi* generates a biomass with improved chemopreventive activity, especially against the proliferation of the aggressive cancer prostate PC3 cells.

These results are worthy of interest, since the bioactivity benefit from co-cultivation does not add any cost compared to mono-algal cultivation. This study experimentally proposes a new microalgal cultivation strategy aiming to boost their application in eco-sustainable biotechnology and paves the way for further investigations to better define its strengths and limits towards potential up-scaling application.

## Figures and Tables

**Table 1:** Growth and biomass yield of the microalgal cultivation.

	Cell Concentration (Cells mL^−1^)	Growth Rate (d^−1^)	Integrated GR (d^−1^)	DW (mg L^−1^)
S.m.	5.73 × 10^5^ ± 1.2 × 10^4^	0.91 ± 0.18	0.82 ± 0.10	97.63 ± 34.17
C.c.	1.77 × 10^5^ ± 5.2 × 10^4^	0.58 ± 0.07	0.36 ± 0.03	76.20 ± 28.39
N.o.	2.31 × 10^6^ ± 1.5 × 10^5^	0.55 ± 0.02	0.54 ± 0.06	36.44 ± 2.04
S.m.	2.48 × 10^5^ ± 4.7 × 10^4^ (S.m.: 57.2%	0.72 ± 0.19	0.59 ± 0.04	122.99 ± 15.59
+				
C.c.	1.86 × 10^5^ ± 3.5 × 10^4^ (C.c.: 42.8%)	0.39 ± 0.12	0.58 ± 0.04	(S.m. 34.2%; C.c. 65.8%)
C.c.	1.80 × 10^5^ ± 3.0 × 10^4^ (C.c.: 36.5%)	0.20 ± 0.10	0.39 ± 0.03	81.87 ± 25.12
+				
N.o.	3.21 × 10^5^ ± 1.0 × 10^5^ (N.o.: 63.5%)	0.17 ± 0.06	0.25 ± 0.02	(C.c. 93.9%; N.o. 6.1%)
S.m.	4.23 × 10^5^ ± 7.7 × 10^4^ (S.m.: 36.8%)	0.45 ± 0.04	0.44 ± 0.03	86.24 ± 14.50
+				
N.o.	7.23 × 10^5^ ± 8.9 × 10^4^ (N.o.: 63.2%)	0.26 ± 0.02	0.35 ± 0.02	(S.m. 85.6%; N.o. 14.2%)

Note: Integrated growth rate represents the growth rate estimated over the exponential phase of growth curve (d^−1^). DW: dry weight of harvested biomass (mg L^−1^). All values are represented as the mean ± SD of three independent experiments. S.m. = *Skeletonema marinoi*, C.c.= *Cyclotella cryptica*, N.o.= *Nannochloropsis oceanica,* S.m. + N.o.= co-cultivation of S.m. and N.o., C.c. + N.o.= co-cultivation of C.c. and N.o., S.m.+ C.c.= co-cultivation of C.c. and S.m. Values are expressed as mean ± SD.

## Data Availability

Data is contained within the article and supplementary material.

## Supplementary Materials

The supplementary materials are available online at https://www.mdpi.com/article/10.3390/antiox10091360/s1.

## References

[1] Rizwan M., Mujtaba G., Memon S.A., Lee K., Rashid N. (2018). Exploring the potential of microalgae for new biotechnology applications and beyond: A review. Renew. Sustain. Energy Rev..

[2] Barra L., Chandrasekaran R., Corato F., Brunet C. (2014). The Challenge of Ecophysiological Biodiversity for Biotechnological Applications of Marine Microalgae. Mar. Drugs.

[3] Galasso C., Gentile A., Orefice I., Ianora A., Bruno A., Noonan D.M., Sansone C., Albini A., Brunet C. (2019). Microalgal Derivatives as Potential Nutraceutical and Food Supplements for Human Health: A Focus on Cancer Prevention and Interception. Nutrients.

[4] Vigani M., Parisi C., Rodríguez-Cerezo E., Barbosa M.J., Sijtsma L., Ploeg M., Enzing C. (2015). Food and feed products from micro-algae: Market opportunities and challenges for the EU. Trends Food Sci. Technol..

[5] Global Microalgae Market (2020). Global Microalgae Market 2020 by Manufacturers, Regions, Type and Application, Forecast to 2025. https://www.marketstudyreport.com/reports/global-microalgae-market-2020-by-manufacturers-regions-type-and-application-forecast-to-2025.

[6] Mobin S., Alam F. (2017). Some promising microalgal species for commercial applications: A review. Energy Procedia.

[7] Novoveská L., Ross M., Stanley M.S., Pradelles R., Wasiolek V., Sassi J.F. (2019). Microalgal Carotenoids: A Review of Production, Current Markets, Regulations, and Future Direction. Mar. Drugs.

[8] Mutanda T., Naidoo D., Bwapwa J.K., Anandraj A. (2020). Biotechnological Applications of Microalgal Oleaginous Compounds: Current Trends on Microalgal Bioprocess Products. Front. Energy Res..

[9] Acien F.G., Fernández J.M., Magán J.J., Molina E. (2012). Production cost of a real microalgae production plant and strategies to reduce it. Biotechnol. Adv..

[10] Blanken W., Cuaresma M., Wijffels R.H., Janssen M. (2013). Cultivation of microalgae on artificial light comes at a cost. Algal Res..

[11] Ruiz J., Olivieri G., de Vree J., Bosma R., Willems P., Reith J.H., Eppink M.H.M., Kleinegris D.M.M., Wijffels R.M., Barbosa M.J. (2016). Towards industrial products from microalgae. Energy Environ. Sci..

[12] Clippinger J., Davis R. (2015). Techno-Economic Analysis for the Production of Algal Biomass via Closed Photobioreactors: Future Cost Potential Evaluated across a Range of Cultivation System Designs.

[13] Williams P.J.B., Laurens L.M.L. (2010). Microalgae as biodiesel & biomass feedstocks: Review & analysis of the biochemistry, energetics & economics. Energy Environ. Sci..

[14] Del Mondo A., Smerilli A., Sané E., Sansone C., Brunet C. (2020). Challenging microalgal vitamins for human health. Microb. Cell Fact..

[15] Del Mondo A., Smerilli A., Ambrosino L., Albini A., Noonan D.M., Sansone C., Brunet C. (2021). Insights into phenolic compound from microalgae: Structural variety and complex beneficial activities from health to nutraceutics. Crit. Rev. Biotechnol..

[16] Sansone C., Brunet C. (2019). Promises and challenges of microalgal antioxidant production. Antioxidants.

[17] Hajinajaf N., Mehrabadi A., Tavakoli O. (2021). Practical strategies to improve harvestable biomass energy yield in microalgal culture: A review. Biomass Bioenergy.

[18] Jacobi A., Steinweg C., Sastre R.R., Posten C. (2012). Advanced photobioreactor LED illumination system: Scale-down approach to study microalgal growth kinetics. Eng. Life Sci..

[19] Rebolledo-Oyarce J., Mejía-López J., García G., Rodríguez-Córdova L., Sáez-Navarrete C. (2019). Novel photobioreactor design for the culture of *Dunaliella tertiolecta*—Impact of color in the growth of microalgae. Bioresour. Technol..

[20] Atta M., Idris A., Bukhari A., Wahidin S. (2013). Intensity of blue LED light: A potential stimulus for biomass and lipid content in fresh water microalgae *Chlorella vulgaris*. Bioresour. Technol..

[21] Darko E., Heydarizadeh P., Schoefs B., Sabzalian M.R. (2014). Photosynthesis under artificial light: The shift in primary and secondary metabolism. Philos. Trans. R. Soc. B Biol. Sci..

[22] Orefice I., Musella M., Smerilli A., Sansone C., Chandrasekaran R., Corato F., Brunet C. (2019). Role of nutrient concentrations and water movement on diatom’s productivity in culture. Sci. Rep..

[23] Smerilli A., Orefice I., Corato F., Ruban A., Brunet C. (2017). Photoprotective and antioxidant responses to light spectrum and intensity variations in the coastal diatom *Skeletonema marinoi*. Environ. Microbiol..

[24] Smerilli A., Balzano S., Maselli M., Blasio M., Orefice I., Galasso C., Sansone C., Brunet C. (2019). Antioxidant and Photoprotection Networking in the Coastal Diatom *Skeletonema marinoi*. Antioxidants.

[25] Padmaperuma G., Kapoore R.V., Gilmour D.J., Vaidyanathan S. (2018). Microbial consortia: A critical look at microalgae co-cultures for enhanced biomanufacturing. Crit. Rev. Biotechnol..

[26] Cho D.H., Ramanan R., Heo J., Lee J., Kim B.H., Oh H.M., Kim H.S. (2015). Enhancing microalgal biomass productivity by engineering a microalgal–bacterial community. Bioresour. Technol..

[27] Hays S.G., Patrick W.G., Ziesack M., Oxman N., Silver P.A. (2015). Better together: Engineering and application of microbial symbioses. Curr. Opin. Biotechnol..

[28] Hutchinson G.E. (1961). The paradox of the plankton. Am. Nat..

[29] Li Z., Wang Q., Ruan X., Cunde P., Jiang D. (2010). Phenolics and Plant Allelopathy. Molecules.

[30] Brown M.R., Jeffrey S.W., Volkman J.K., Dunstan G. (1997). Nutritional properties of microalgae for mariculture. Aquaculture.

[31] Traller J.C., Cokus S.J., Lopez D.A., Gaidarenko O., Smith S.R., McCrow J.P., Gallaher S.D., Podell S., Thompson M., Cook O. (2016). Genome and methylome of the oleaginous diatom *Cyclotella cryptica* reveal genetix flexibility toward a high lipid phenotype. Biotechnol. Biofuels.

[32] Xiao Y., Zhang J., Cui J., Feng Y., Cui Q. (2013). Metabolic profiles of *Nannochloropsis oceanica* IMET1 under nitrogen-deficiency stress. Bioresour. Technol..

[33] Zanella L., Vianello F. (2020). Microalgae of the genus *Nannochloropsis*: Chemical composition and functional implications for human nutrition. J. Funct. Food.

[34] Floegel A., Kim D., Chung S., Koo S.I., Chun O.K. (2011). Comparison of ABTS/DPPH assays to measure antioxidant capacity in popular antioxidant-rich US foods. J. Food Compos. Anal..

[35] Chandrasekaran R., Barra L., Carillo S., Caruso T., Corsaro L., Dal Piaz F., Graziani G., Corato F., Pepe D., Manfredonia A. (2014). Light modulation of biomass and macromolecular composition of the diatom *Skeletonema marinoi*. J. Biotechnol..

[36] Orefice I., Chandrasekaran R., Smerilli A., Corato F., Carillo S., Caruso T., Corsaro M.M., Dal Piaz F., Ruban A., Brunet C. (2016). Light-induced changes in the photosynthetic physiology and biochemistry in the diatom *Skeletonema marinoi*. Algal Res..

[37] Brunet C., Corato F. (2015). PhoLia: A Light System for Aquatic Organisms. European Patent.

[38] Cheng D., Li D., Yuan Y., Zhou L., Li X., Wu T., Wang L., Quanyu Z., Wei W., Yuhan S. (2017). Improving carbohydrate and starch accumulation in *Chlorella* sp. AE10 by a novel two-stage process with cell dilution. Biotechnol. Biofuels.

[39] Desjardins P., Hansen J.B., Allen M. (2009). Microvolume spectrophotometric and fluorometric determination of protein concentration. Curr. Protoc. Protein Sci..

[40] Ryckebosch E., Muylaert K., Foubert I. (2011). Optimization of an Analytical Procedure for Extraction of Lipids from Microalgae. J. Am. Oil Chem. Soc..

[41] Araújo L.B.D.C., Silva S.L., Galvão M.A.M., Ferreira M.R.A., Araújo E.L., Randau K., Soares L.A.L. (2013). Total phytosterol content in drug materials and extracts from roots of *Acanthospermum hispidum* by UV-VIS spectrophotometry. Rev. Bras. Farmacogn..

[42] Nour V., Trandafir I., Cosmulescu S. (2012). HPLC Determination of Phenolic Acids, Flavonoids and Juglone in Walnut Leaves. J. Chromatogr. Sci..

[43] Brunet C., Casotti R., Vantrepotte V. (2008). Phytoplankton diel and vertical variability in photobiological responses at a coastal station in the Mediterranean Sea. J. Plankton Res..

[44] Lee K.W., Lee H.J., Surh Y.-J., Lee C.Y. (2003). Vitamin C and cancer chemoprevention: Reappraisal. Am. J. Clin. Nutr..

[45] Ilie P.C., Stefanescu S., Smith L. (2020). The role of vitamin D in the prevention of coronavirus disease 2019 infection and mortality. Aging Clin. Exp. Res..

[46] Weston A.L., Mathesius U. (2013). Flavonoids: Their Structure, Biosynthesis and Role in the Rhizosphere, Including Allelopathy. J. Chem. Ecol..

[47] Bacellar Mendes L.B., Vermelho A.B. (2013). Allelopathy as a potential strategy to improve microalgae cultivation. Biotechnol. Biofuels.

[48] Lu Y., Tarkowská D., Turečková V., Luo T., Xin Y., Li J., Wang Q., Jiao N., Strnad M., Xu J. (2014). Antagonistic roles of abscisic acid and cytokinin during response to nitrogen depletion in oleaginous microalga *Nannochloropsis oceanica* expand the evolutionary breadth of phytohormone function. Plant J..

[49] Long M., Tallec K., Soudant P., Le Grand F., Donval A., Lambert C., Sarthou G., Jolley D.F., Hégaret H. (2018). Allelochemicals from *Alexandrium minutum* induce rapid inhibition of metabolism and modify the membranes from *Chaetoceros muelleri*. Algal Res..

[50] Menon M., Maramag C., Malhotra R.K., Seethalakshmi L. (1998). Effect of vitamin C on androgen independent prostate cancer cells (PC3 and Mat-Ly-Lu) in vitro: Involvement of reactive oxygen species-effect on cell number, viability and DNA synthesis. Cancer Biochem. Biophys..

[51] Morley S., Thakur V., Danielpour D., Parker R., Arai H., Atkinson J., Barnholtz-Sloan J., Klein E., Manor D. (2010). Tocopherol transfer protein sensitizes prostate cancer cells to vitamin E. J. Biol. Chem..

[52] Zheng Y., Trivedi T., Cy Lin R., Fong-Yee C., Nolte R., Manibo J., Chen Y., Hossain M., Horas K., Dunstan C. (2017). Loss of the vitamin D receptor in human breast and prostate cancers strongly induces cell apoptosis through downregulation of Wnt/β-catenin signaling. Bone Res..

[53] Cussenot O., Berthon P., Berger R., Mowszowicz I., Faille A., Hojman F., Teillac P., Le Duc A., Calvo F. (1991). Immortalization of human adult normal prostatic epithelial cells by liposomes containing large T-SV40 gene. J. Urol..

[54] Sullivan C.S., Pipas J.M. (2002). T antigens of simian virus 40: Molecular chaperones for vital replication and tumorigenesis. Microbiol. Mol. Biol. Rev..

[55] Bettuzzi S., Scorcioni F., Astancolle S., Davalli P., Scaltriti M., Corti A. (2002). Clusterin (SGP-2) transient overexpression decreases proliferation rate of SV40-immortalized human prostate epithelial cells by slowing down cell cycle progression. Oncogene.

[56] Camini F.C., da Silva C.C., Trindade Almeida L., de Brito Magalhães L. (2017). Implications of oxidative stress on viral pathogenesis. Arch. Virol..

[57] Fiorino S., Bacchi-Reggiani L., Sabbatani S., Grizzi F., di Tommaso L., Masetti M., Fornelli A., Bondi A., de Biase D., Visani M. (2014). Possible role of tocopherols in the modulation of host microRNA with potential antiviral activity in patients with hepatitis B virus-related persistent infection: A systematic review. Br. J. Nutr..

[58] Rahman M.M., Mosaddik A., Alam A.K. (2021). Traditional foods with their constituent’s antiviral and immune system modulating properties. Heliyon.

[59] Sobue S., Mizutani N., Aoyama Y., Kawamoto Y., Suzuki M., Nozawa Y., Ichihara M., Murate T. (2016). Mechanism of paclitaxel resistance in a human prostate cancer cell line, PC3-PR, and its sensitization by cabazitaxel. Biochem. Biophys. Res. Commun..

[60] Wissing M.D., Dadon T., Kim E., Piontek K.B., Shim J.S., Kaelber N.S., Liu J.O., Kachhap S.K., Nelkin B.D. (2014). Small-molecule screening of PC3 prostate cancer cell identifies tilorone dihydrochloride to selectively inhibit cell growth based on cyclin-dependent kinase 5 expression. Oncol. Rep..

[61] Méndez-López L.F., Caboni P., Arredondo-Espinoza E., Carrizales-Castillo J.J.J., Balderas-Rentería I., Camacho-Corona M.d.R. (2021). Bioassay-guided identification of the antiproliferative compounds of *Cissus trifoliate* and the transcriptomic effect of resveratrol in prostate cancer PC3 cells. Molecules.

[62] Ferdous U.T., Yusof Z.N.B. (2021). Medicinal prospect of antioxidants from algal sources in cancer therapy. Front. Pharmacol..

