# Supplementary material for: Microalgal Co-Cultivation Prospecting to Modulate Vitamin and Bioactive Compounds Production
Source: Antioxidants (Basel). 2021 Aug 26;10(9):1360. doi: 10.3390/antiox10091360 (PMC8468856; doi:10.3390/antiox10091360)
Supplement: Supplementary file 1 [file antioxidants-10-01360-s001.zip › antioxidants-1294698-proof done supp/Figure S2_SM.docx]

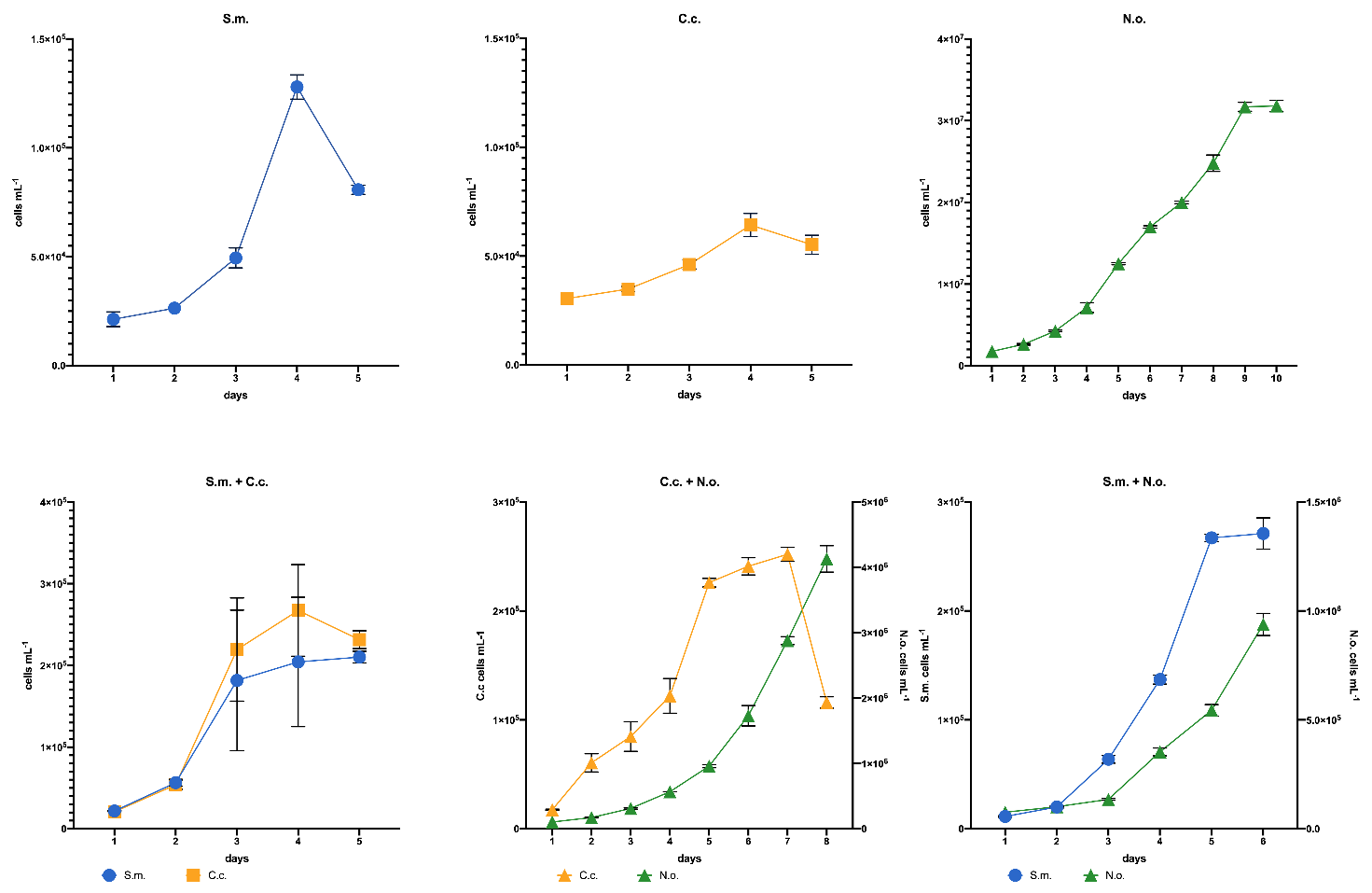


**Figure S2.** Growth curves of S.m. = *Skeletonema marinoi*, C.c.= *Cyclotella cryptica*, N.o.= *Nannochloropsis oceanica*, S.m.+ N.o.= co-cultivation of S.m. and N.o., C.c. + N.o.= co-cultivation of C.c. and N.o., S.m.+ C.c.= co-cultivation of C.c. and S.m. All values are represented as the mean ± SD of three independent experiments.
