# Supplementary material for: Microalgal Co-Cultivation Prospecting to Modulate Vitamin and Bioactive Compounds Production
Source: Antioxidants (Basel). 2021 Aug 26;10(9):1360. doi: 10.3390/antiox10091360 (PMC8468856; doi:10.3390/antiox10091360)
Supplement: Supplementary file 1 [file antioxidants-10-01360-s001.zip › antioxidants-1294698-proof done supp/Figure S3_SM.docx]

**
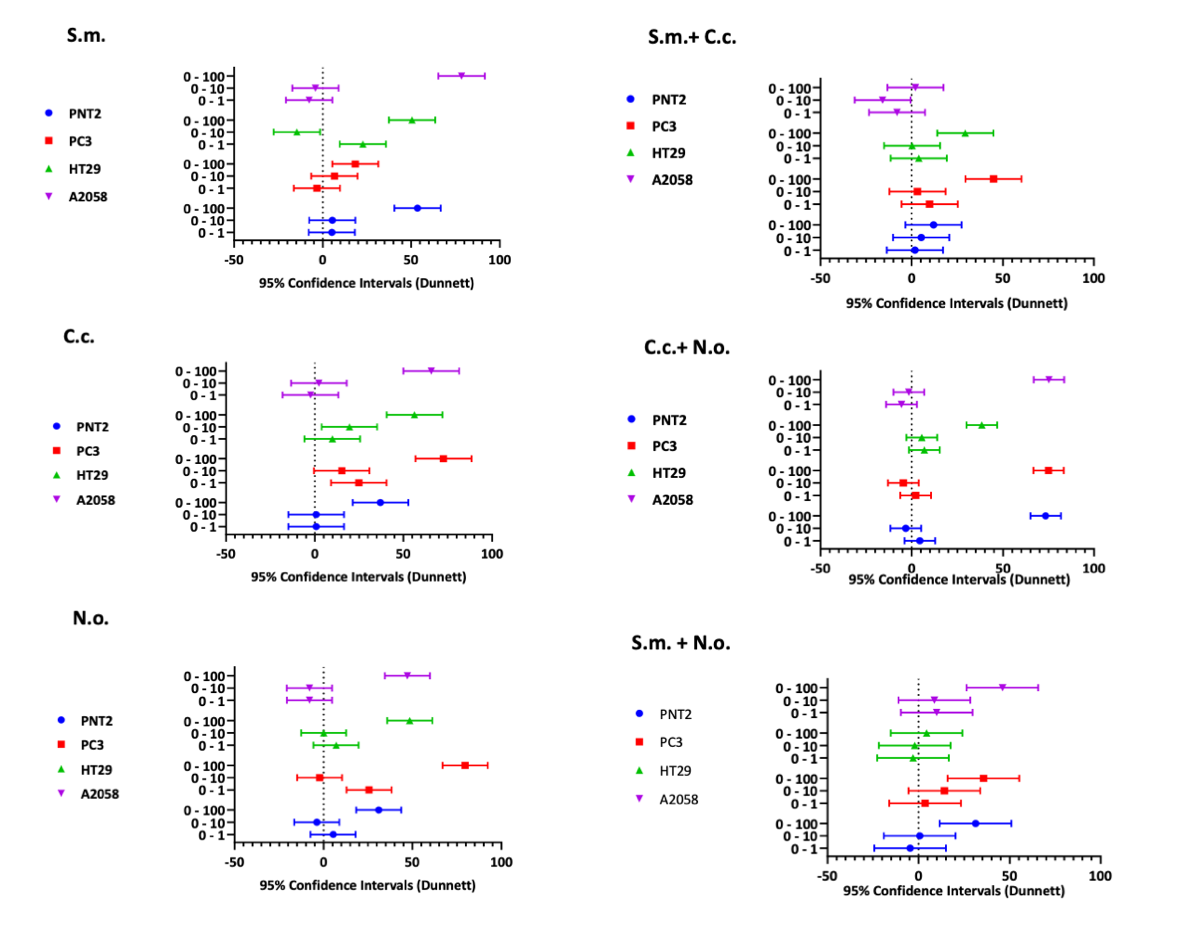
**

**Figure S3.** Confidence intervals calculated for MTT assay on the PNT2, PC3, HT29 and A2058 cell lines through Dunnett test: S.m. = *Skeletonema marinoi*, C.c.= *Cyclotella cryptica*, N.o.= *Nannochloropsis oceanica*, S.m. + N.o.= co-cultivation of S.m. and N.o., C.c. + N.o.= co-cultivation of C.c. and N.o., S.m.+ C.c.= co-cultivation of C.c. and S.m.
