# Supplementary material for: Microalgal Co-Cultivation Prospecting to Modulate Vitamin and Bioactive Compounds Production
Source: Antioxidants (Basel). 2021 Aug 26;10(9):1360. doi: 10.3390/antiox10091360 (PMC8468856; doi:10.3390/antiox10091360)
Supplement: Supplementary file 1 [file antioxidants-10-01360-s001.zip › antioxidants-1294698-proof done supp/Table S1_SM.docx]

**Table S1.** The three selected algal species: *Skeletonema marinoi (S.m.), Cyclotella cryptica (C.c.),* and *Nannochloropsis oceanica (N.o.).*

| **species** | **strain** | **class** | **habitat** | **size** | **morphology** | **application** |
| --- | --- | --- | --- | --- | --- | --- |
| *Skeletonema marinoi* | *CCMP 2092* | Bacillariophyceae | marine, coastal | 7-11 µm | centric, chain forming | feed in aquaculture  [31] |
| *Cyclotella*  *cryptica* | *CCMP*  *332* | Bacillariophyceae | marine, brackish | 3-5 µm | centric, single cell | candidate for PUFA production  [32] |
| *Nannochloropsis oceanica* | *CCMP 1779* | Eustigmatophyceae | marine | 2-3 µm | single cell | candidate for PUFA production  [33] |

References:

30. Brown, M.R.; Jeffrey, S.W.; Volkman, J.K.; Dunstan, G. Nutritional properties of microalgae for mariculture. *Aquaculture* **1997**, *1-4*, 315–331, doi:10.1016/S0044-8486(96)01501-3.

31. Traller, J.C.; Cokus, S.J.; Lopez, D.A.; Gaidarenko, O.; Smith, S.R.; McCrow, J.P.; Gallaher, S.D.; Podell, S.; Thompson, M.; Cook, O. Genome and methylome of the oleaginous diatom *Cyclotella cryptica* reveal genetix flexibility toward a high lipid phenotype. *Biotechnol. Biofuels* **2016**, *9*, 258, doi:10.1186/s13068-016-0670-3.

32. Xiao, Y.; Zhang, J.; Cui, J.; Feng, Y.; Cui, Q. Metabolic profiles of *Nannochloropsis oceanica* IMET1 under nitrogen-deficiency stress. *Bioresour. Technol*. **2013**, *130*, 731–738, doi:10.1016/j.biortech.2012.11.116.
