# Supplementary material for: Microalgal Co-Cultivation Prospecting to Modulate Vitamin and Bioactive Compounds Production
Source: Antioxidants (Basel). 2021 Aug 26;10(9):1360. doi: 10.3390/antiox10091360 (PMC8468856; doi:10.3390/antiox10091360)
Supplement: Supplementary file 1 [file antioxidants-10-01360-s001.zip › antioxidants-1294698-proof done supp/Table S2_SM.docx]

**Table S2.** List of antibodies used for vitamins’ determination applying competitive ELISA assay.

| **Brand** | **Code** | **Target** | **Host** | **Clonality** |
| --- | --- | --- | --- | --- |
| Creative Diagnostics | CABT-B8962 | Anti-Vitamin A | Rabbit | Polyclonal |
| Cloud Clone | PAD053Ge01 | Anti-Vitamin B_1_ | Rabbit | Polyclonal |
| Cloud Clone | PAD054Ge01 | Anti-Vitamin B_2_ | Rabbit | Polyclonal |
| Cloud Clone | PAA916Ge01 | Anti-Vitamin B_6_ | Rabbit | Polyclonal |
| Creative Diagnostics | DPATB-H83238 | Anti-Vitamin B_12_ | Rabbit | Polyclonal |
| Cloud Clone | PAA913Ge01 | Anti-Vitamin C | Rabbit | Polyclonal |
| Cloud Clone | PAA921Ge01 | Anti-Vitamin D_2_ | Rabbit | Polyclonal |
| Cloud Clone | PAA920Ge01 | Anti-Vitamin D_3_ | Rabbit | Polyclonal |
| Creative Diagnostics | DPAB-DC3974 | Anti-Alpha-Tocopherol | Rabbit | Polyclonal |
| Creative Diagnostics | DPAB-DC4159 | Anti-D-Biotin | Rabbit | Polyclonal |
