# Supplementary material for: Microalgal Co-Cultivation Prospecting to Modulate Vitamin and Bioactive Compounds Production
Source: Antioxidants (Basel). 2021 Aug 26;10(9):1360. doi: 10.3390/antiox10091360 (PMC8468856; doi:10.3390/antiox10091360)
Supplement: Supplementary file 1 [file antioxidants-10-01360-s001.zip › antioxidants-1294698-proof done supp/Table S3_SM.docx]

**Table S3.** The main phenolic compounds detected in S.m. and C.c. Data in µg mg^-1^ DW. All values are represented as the mean ± SD of three independent experiments. S.m. = *Skeletonema marinoi*; C.c.= *Cyclotella cryptica*.

|  | **Gallic acid** | **Sinapic acid** | **p-coumaric acid** | **Rutin** | **Daidezein** | **Genistein** | **Apigenin** |
| --- | --- | --- | --- | --- | --- | --- | --- |
| S.m. | 0.208 ± 0.071 | 0.339 ± 0.104 | n.d. | 0.291 ± 0.143 | 0.259 ± 0.129 | 0.396 ± 0.081 | n.d. |
| C.c. | 0.070 ± 0.010 | 0.023 ± 0.011 | 0.057 ± 0.030 | n.d. | 0.470 ± 0.168 | 0.061 ± 0.010 | 0.560 ± 0.173 |
